# Sex, Age, and Comorbidities Are Associated with SARS-CoV-2 Infection, COVID-19 Severity, and Fatal Outcome in a Mexican Population: A Retrospective Multi-Hospital Study

**DOI:** 10.3390/jcm12072676

**Published:** 2023-04-03

**Authors:** Maria Elena Camacho Moll, Viviana Leticia Mata Tijerina, Beatriz Silva Ramírez, Katia Peñuelas Urquides, Laura Adiene González Escalante, Brenda Leticia Escobedo Guajardo, Jorge Eleazar Cruz Luna, Roberto Corrales Pérez, Salvador Gómez García, Mario Bermúdez de León

**Affiliations:** 1 Laboratory of Molecular Biology, Northeast Biomedical Research Centre, Mexican Social Security Institute, Monterrey 64720, Mexico; 2 Laboratory of Immunogenetics, Northeast Biomedical Research Centre, Mexican Social Security Institute, Monterrey 64720, Mexico; 3 Laboratory of Molecular Microbiology, Northeast Biomedical Research Centre, Mexican Social Security Institute, Monterrey 64720, Mexico; 4 Laboratory of Molecular Research of Diseases, Northeast Biomedical Research Centre, Mexican Social Security Institute, Monterrey 64720, Mexico; 5 Medical Epidemiological Assistance Coordination of the State of Nuevo León, Mexican Social Security Institute, Monterrey 64000, Mexico

**Keywords:** COVID-19, SARS-CoV-2, association, severity, death, Mexico

## Abstract

People with comorbidities and the male sex are at a higher risk of developing severe COVID-19. In the present study, we aim to investigate the associated factors for infection, severity, and death due to COVID-19 in a population from Nuevo León, México. Epidemiological COVID-19 data were collected from 65 hospitals from December 2020 to May 2022. A total of 75,232 cases were compiled from which 25,722 cases were positive for SARS-CoV-2. Male sex, older age, diabetes, obesity, and hypertension were associated with infection. In addition to the above-mentioned factors, renal disease, cardiovascular disease, and immunosuppression were found to be associated with increased COVID-19 severity. These factors, as well as neurological diseases, are also associated with death due to COVID-19. When comparing the different variants of SARs-CoV-2, the variant B1.1.519 increased the probability of death by 2.23 times compared to the AY.20 variant. Male sex, older age, diabetes, obesity, and hypertension are associated with SARS-CoV-2 infection, severity, and death. Along with the aforementioned comorbidities, renal disease, cardiovascular disease, and immunosuppression are also associated with severity and death. Another factor associated with death is the presence of neurological disease. The SARS-CoV-2 B1.1.519 variant increases the odds of death compared to the SARS-CoV-2 AY.20 variant.

## 1. Introduction

The world is recently recovering from the COVID-19 pandemic, a disease that originated in the seafood market located in Wuhan, Hubei, China. Since the first case in 2019 up to October 2022, more than 6 million lives have been lost and there are 755,703,002 COVID-19 confirmed cases [1]. The virus responsible for this disease is of the coronavirus type and is now termed SARS-CoV-2. Patients with the mild disease most commonly displayed a cough, hyposmia, and sputum with fever only present in around 11% of patients [2]; patients with severe COVID-19 displayed dyspnea and commonly present comorbidities [3]. Patients with the critical disease had respiratory difficulties, acute cardiac lesions, and multiple organ failure [3].

At the beginning of the pandemic, male sex, age, and several comorbidities were associated with an increased risk of severe COVID-19, such as hypertension, diabetes, renal disease, chronic obstructive pulmonary disease (COPD), autoimmune diseases, and cancer [4,5,6,7,8]. In Mexico, COVID-19 vaccination started in December 2020 and the administration was in stages according to priority groups with vaccines varying in type upon availability [9]. By June 2022, a total of 209,673,612 vaccines were administered [10]. Furthermore, by that time, 91% of the population over 18 years old had at least one dose and a booster dose was already being administered to the same population group [10]. By June 2022, one dose of the COVID-19 vaccine had already been administered to 55% of adolescents (12 to 17 years of age) [10]. In the present study, we analyzed epidemiological data of a population from the northeast of Mexico, where we report the associated factors of infection, severity, and outcome of COVID-19 disease.

## 2. Materials and Methods

### 2.1. Database and Data Depuration

**Figure 1:**
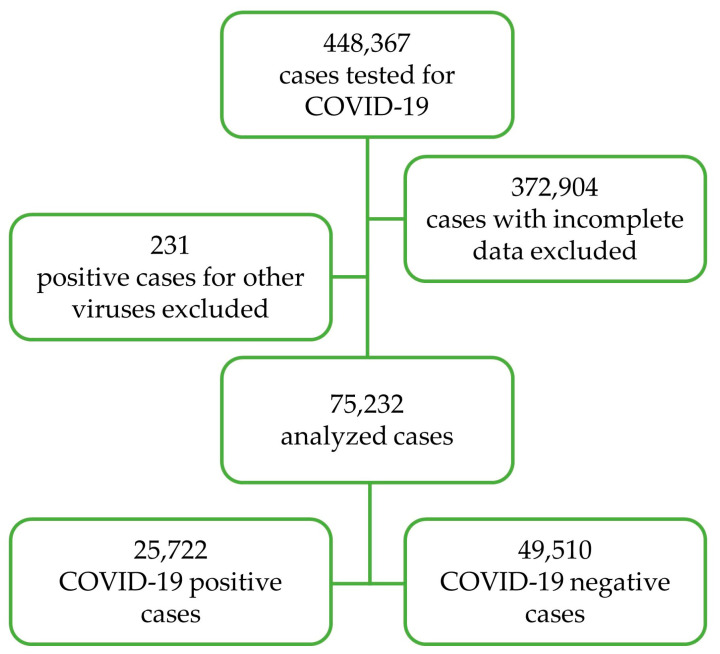
Schematic representation of data depuration.

A database containing information about the patients that required COVID-19 testing from 18 December 2020 to 7 April 2022 was obtained. The database comprised a total of 448,367 cases from 65 hospitals distributed in Nuevo León, Mexico. Out of 448,367 cases, 25,787 (5.8%) were COVID-19 positive, determined using quantitative polymerase chain reaction (qPCR), and the variant determination was performed by the Instituto de Diagnóstico y Referencia Epidemiológicos (InDRE), Secretaria de Salud, Mexico, as approved by the World Health Organization [11]. Cases with incomplete data and positive cases for other viruses were excluded from the analysis. A total of 75,232 cases were analyzed (Figure 1). 

### 2.2. Statistical Analysis

Categorical variables were described in frequencies. For non-categorical variables, means and standard deviation were calculated. The association of SARS-CoV-2 infection, severity and outcome to sex, age, comorbidities, and tobacco smoking was evaluated using the chi-square test. This was followed by stepwise multivariate logistic regressions for infection and outcome analyses and stepwise multivariate logistic ordinal regression models for severity analysis. In all models, the independent variables were sex, age, comorbidities, and smoking. The severity variable was constructed by categorizing COVID-19-positive patients into mild, severe (diagnosed pneumonia), and critical (required admission to an Intensive Care Unit [ICU] and/or endotracheal intubation). 

## 3. Results

### 3.1. Characteristics of the Studied Population

A total of 75,232 cases were analyzed, of which 87% were reported as unvaccinated. The mean age was 43.6 ± 20.6 years. Comorbidities were present in 31.5% of the population where hypertension, obesity, and diabetes were the most common. Patients with two comorbidities represented 9.6%, whereas patients with more than two comorbidities represented 6.3%. Smokers represented 4.3% of the population. There were 25,755 (34.2%) COVID-19-positive patients with a positive PCR rate of 33.7% in 2021 and 38.2% in 2022. The mild disease was developed by 90.7%, 8.2% developed a moderate disease, and 1.2% developed a critical disease. Of the COVID-19-positive patients, 89.3% recovered, 10% died, 0.2% opted for voluntary discharge, and 0.5% were referred to other clinics (Table 1). The death rate was 17.8% for 2021 and 12.8% for 2022. Variant information was only available for 396 cases; details can be found in Table S1. The most commonly reported SARS-CoV2 variants were AY.20 (31.8%), followed by B.1.1.519 (15.7%) and AY.26 (14.7%).

### 3.2. Associated Factors with SARS-CoV-2 Infection

Several factors are associated to SARS-CoV-2 infection, such as male sex, older age, having diabetes, obesity, hypertension, and presenting two comorbidities (Table 2). A reduction in the odds of infection within patients with renal disease, COPD, other not disclosed comorbidities, and more than two comorbidities was observed. Tobacco smokers also showed a decreased probability of infection (Table 2). 

### 3.3. Associated Factors with Critical COVID-19

A multivariate analysis was performed to determine factors associated with severe/critical COVID-19 patients, who were hospitalized patients that required either ICU attention or endotracheal intubation. Comparable to factors associated with infection, a group of variables, such as male sex, older age, diabetes, obesity, hypertension, cardiovascular disease, renal disease, immunosuppression, and other comorbidities, were associated with severe/critical COVID-19. Tobacco smoking shows significantly reduced odds of severe/critical COVID-19 (Table 3). 

### 3.4. Associated Factors with Death as an Outcome of SARS-CoV-2 Infection

An increased probability of death as an outcome of SARS-CoV-2 infection was found in the male sex, older age, patients with hypertension, obesity, diabetes, cardiovascular disease, renal disease, immunosuppression, neurological diseases, other non-described comorbidities, and having two or more comorbidities. Interestingly, tobacco smoking reduced the odds of death (Table 4).

### 3.5. COVID-19 Variants and Their Association with Death as an Outcome of COVID-19

SARS-CoV-2 variant information and multivariate regression analysis estimated an increase of 2.23 times the probability of death when the infecting variant was B.1.1.519 compared to AY.20 (Table 5).

## 4. Discussion

The current manuscript describes the clinical manifestations of COVID-19 in the northeastern Mexican population, where analyses were performed to determine the factors associated with infection, as well as the severity of COVID-19, and death as an outcome of this disease from an epidemiological database from 2020 to 2021. The statistical analysis confirms the association with increased infection odds, severity, and death probability in patients with comorbidities, such as obesity, diabetes, COPD, kidney disease, cardiovascular disease, immunosuppression, and neurological disease, as well as demographic characteristics, such as male sex and older age. 

A recent analysis described factors associated with hospitalization and death in SARS-CoV-2-positive patients, where comorbidities were pointed out as associated factors. However, specific comorbidities were not further described [12]. In the present study, we considered SARS-CoV-2-positive patients to be only those with a confirmed PCR test, whereas Loza and colleagues (2022) considered positive to be those with a positive antigen test or a positive result ruled by epidemiologic association, with an increased probability of analyzing COVID-19 negative patients as positives [13,14].

Particularly, a recent study reported male sex, age, and comorbidities as risk factors for SARS-CoV-2 infection, pneumonia, intubation, and death in data collected from 11 February 2020 to 24 September 2020 with a total of 35,476 positive cases from a Mexican northeastern population [15]. By comparing periods of time analyzed by Hid-Cordero and colleagues (2021), the predominant SARS-CoV-2 variant was B.1, B.1.1, and B.1.1222, whereas, during the period studied in the present investigation, the predominant variants were delta (AY.20), followed by B.1.1.519.

Among the associated factors to infection, severity, and death; sex has been one of the characteristics of the population previously described, where males have more prevalence and mortality due to COVID-19 [16,17]. Some theories have come forward to explain sex differences regarding the immune system, where women experienced a stronger immune response compared to men [17]. Sexual hormones may also have a role in COVID-19 severity since it has been shown that estrogens are immunostimulants, whereas testosterone has immunosuppressive effects [18]. Furthermore, the ACE-2 receptor, which is responsible for the binding and internalization of the viral particles of SARS-CoV2 [19], can be induced by testosterone in lung cells, whereas estrogens demonstrated a trend toward a reduction in ACE-2 in lung cells [20].

Another factor, which has been previously associated with severe COVID-19 is tobacco smoking [6]. Smoking was very interesting to investigate in the Mexican population given that according to data from 2016, there were more smokers in the male population compared to females, with 36.6% and 15.5%, respectively [21]. Therefore, this was a lifestyle condition, which could be expected to predispose Mexican men to severe COVID-19. However, smoking was not positively associated in any of the analyses that we performed and the contrary phenomenon was observed, where smokers had a decreased probability of infection, severity, and death; this result has also been reported before [4,12,15]. An explanation for this could be that nicotine can have immunomodulatory and anti-inflammatory effects. Furthermore, tobacco use increases nitric oxide production in the lung which results in a reduction in viral replication and impaired viral entry into the host cells [22]. Within the renin-angiotensin system (RAS), ACE-2 can be found and nicotine can increase angiotensin-I, which in turn, causes a reduction in ACE-2 in the lung; this has been demonstrated in animal models [23]. Nicotine has been suggested as a therapeutic agent to prevent SARS-CoV-2 infection [24]. 

Old age has also been associated with severe COVID-19 [25]. The COVID-19 lethality rate increases with age, and this has been demonstrated in studies from different countries [26,27,28,29]. In the present study, this association is confirmed, and older age is associated with an increased risk of infection, critical COVID-19, and risk of death.

Recent studies have reported an association between COVID-19 severity and comorbidities, such as diabetes [30,31], hypertension [31,32], kidney disease [5,31,33,34], COPD [6,7,31,35,36,37], immune, and chronic inflammatory diseases, such as arthritis and rheumatic diseases [38], obesity [39], among others. Obesity is a major public health problem and according to the World Health Organization, obesity has increased three times from 1975 to 1997, and in 2020, 1.9 million adults were overweight and 650 million were obese [40]. In Mexico, obesity is an important concern, as in 2018, it was reported that in children aged 5–11, 18.1% were overweight and 17.5% were obese, whereas in adults 36.6% of women were overweight and 40.2% of women were obese, 42.5% of men were overweight and 30.5% of men were obese, which adds up to more than 70% of the adult men and women population [41].

We have also collected information about variants and their association with death as an outcome of COVID-19. We have shown that there is an increased predisposition to death in people infected with the B.1.1.519 SARS-CoV-2 variant, which has been previously shown [42].

Some limitations of this study consisted of the fact that many data were not considered due to incomplete or wrong information. This may be due to the saturation of hospitals and the prioritization of patient care during the emergency, rather than the data collection. There was no data analysis performed in the current study regarding vaccination. However, only 13% of the studied population reported at least one dose, which is very low compared to the country’s vaccination reports. This same situation is noticeable regarding comorbidity reports, given that only 8% of the studied population reported obesity and Mexico has one of the highest obesity rates. Another limitation is the registration of variant information, and this could be due to delays in protocol validations and algorithms established by the national epidemiological surveillance system, which determines that only certain samples are genotyped. 

## 5. Conclusions

Sex, age, and comorbidities, such as diabetes, obesity, and hypertension are factors associated with SARS-CoV-2 infection. In addition to these factors, renal, cardiovascular, and immune diseases are associated with increased severity and death. Neurological disease is associated with death due to COVID-19. Compared to the SARS-CoV-2 AY.20 variant, the B.1.1.519 variant significantly increased death as an outcome.

## Figures and Tables

**Table 1:** Sociodemographic and other characteristics of the population studied (*n* = 75,232).

	*n* (%)
Sex	
Female	38,876 (51.7)
Male	36,356 (48.3)
Years of age	
0–9	3644 (4.8)
10–19	3026 (4.0)
20–29	14,392 (19.1)
30–39	13,355 (17.8)
40–49	12,263 (16.3)
50–59	10,988 (14.6)
≥60	17,564 (23.3)
Morbidities and comorbidities	
No reported comorbidities	51,503 (68.5)
Hypertension	3147 (4.2)
Obesity	2616 (3.5)
Diabetes	2501 (3.3)
Asthma	725 (1.0)
Cardiovascular disease	241 (0.3)
Renal disease	235 (0.3)
HIV	170 (0.2)
Immunosuppression	144 (0.2)
COPD	143 (0.2)
Cancer	103 (0.1)
Neurological disease	52 (0.1)
Hepatic disease	29 (0.04)
Tuberculosis	19 (0.03)
Hemolytic anemia	3 (0.003)
Other	1686 (2.2)
Two comorbidities	7207 (9.6)
More than two comorbidities	4708 (6.3)
Tobacco smokers	3198 (4.3)
COVID-19	
Negative	49,510 (65.6)
Positive	25,722 (34.2)
Severity of the disease	
Mild	23,317 (90.7)
Moderate	2106 (8.2)
Critical	299 (1.2)
Outcome	
Recovery	67,196 (89.3)
Death	7494 (10.0)
Voluntary discharge	173 (0.2)
Referred to another medical unit	369 (0.5)

HIV—Human Immunodeficiency Virus, COPD—Chronic Obstructive Pulmonary Disease.

**Table 2:** Associated factors with SARS-CoV-2 infection (*n* = 75,232).

		SARS-CoV-2 PCR Test Result		
	Total	Negative	Positive	OR (CI 95%)
	*n* (%)	*n* (%)	*n* (%)	
Sex				
Female	38,876 (51.7)	26,432 (53.4)	12,444 (48.4)	1.00
Male	36,356 (48.3)	23,078 (46.6)	13,278 (51.6)	1.27 (1.23, 1.31) ***
Age				
0–9	3644 (4.8)	3158 (6.4)	486 (1.9)	1.00
10–19	3026 (4.0)	2211 (4.5)	815 (3.2)	2.42 (2.14, 2.74) ***
20–29	14,392 (19.1)	9339 (18.9)	5053 (19.6)	3.55 (3.20, 3.93) ***
30–39	13,355 (17.8)	8424 (17.0)	4931 (19.2)	3.78 (3.41, 4.19) ***
40–49	12,263 (16.3)	7601 (15.4)	4662 (18.1)	3.91 (3.52, 4.33) ***
50–59	10,988 (14.6)	6893 (13.9)	4095 (15.9)	3.74 (3.37, 4.15) ***
≥60	17,564 (23.3)	11,884 (24.0)	5680 (22.1)	3.00 (2.71, 3.32) ***
Morbidities and comorbidities				
No reported comorbidities	51,503 (68.5)	34,348 (69.4)	17,155 (66.7)	1.00
Diabetes	2501 (3.3)	1494 (3.0)	1007 (3.9)	1.28 (1.18, 1.39) ***
Obesity	2616 (3.5)	1429 (2.9)	1187 (4.6)	1.56 (1.44, 1.69) ***
Hypertension	3147 (4.2)	1869 (3.8)	1278 (5.0)	1.34 (1.24, 1.44) ***
Asthma	725 (1.0)	509 (1.0	216 (0.8)	0.88 (0.75, 1.03)
Cardiovascular disease	241 (0.3)	177 (0.4)	64 (0.2)	0.81 (0.60, 1.08)
Renal disease	235 (0.3)	173 (0.3)	62 (0.2)	0.70 (0.53, 0.94) *
HIV	170 (0.2)	117 (0.2)	53 (0.2)	0.76 (0.55, 1.05)
Immunosuppression	144 (0.2)	99 (0.2)	45 (0.2)	1.04 (0.73, 1.49)
COPD	143 (0.2)	109 (0.2)	34 (0.1)	0.66 (0.45, 0.98) *
Cancer	103 (0.1)	74 (0.1)	29 (0.1)	0.86 (0.56, 1.32)
Neurological disease	52 (0.1)	39 (0.1)	13 (0.1)	0.81 (0.43, 1.54)
Hepatic disease	29 (0.0)	20 (0.0)	9 (0.0)	0.83 (0.37, 1.81)
Tuberculosis	19 (0.0)	14 (0.1)	13 (0.1)	0.65 (0.24, 1.82)
Hemolytic anemia	3 (0.0)	2 (0.0)	1 (0.0)	0.98 (0.09, 10.83)
Other	1686 (2.2)	1215 (2.5)	471 (1.8)	0.86 (0.77, 0.96) **
Two comorbidities	7207 (9.6)	4558 (9.2)	2649 (10.3)	1.17 (1.11, 1.24) ***
More than two comorbidities	4708 (6.3)	3264 (6.6)	1444 (5.6)	0.90 (0.84, 0.97) **
Tobacco smokers	3198 (4.3)	2148 (4.3)	1050 (4.1)	0.82 (0.75, 0.88) ***

HIV—Human Immunodeficiency Virus, COPD—Chronic Obstructive Pulmonary Disease. OR—Odds ratio, CI—Confidence interval. Data were analyzed using stepwise multivariate logistic regression. * *p* < 0.05; ** *p* < 0.01; *** *p* < 0.001.

**Table 3:** Associated factors with critical COVID-19 (*n* = 25,722).

		COVID-19 Severity			
	Total	Mild	Moderate	Severe/Critical	OR (CI 95%)
	*n* (%)	*n* (%)	*n* (%)	*n* (%)	
Sex					
Female	12,444 (48.4)	11,405 (48.9)	911 (43.3)	128 (42.8)	1.00
Male	13,278 (51.6)	11,912 (51.1)	1195 (56.7)	171 (57.2)	1.27 (1.16, 1.39) ***
Age					
0–9	486 (1.9)	464 (2.0)	20 (0.9)	2 (0.7)	1.00
10–19	815 (3.2)	796 (3.4)	16 (0.8)	3 (1.0)	0.52 (0.28, 0.96) *
20–29	5053 (19.6)	4935 (21.2)	103 (4.9)	15 (5.0)	0.52 (0.33, 0.83) **
30–39	4931 (19.2)	4678 (20.1)	211 (10.0)	42 (14.0)	1.15 (0.73, 1.79)
40–49	4662 (18.1)	4361 (18.7)	258 (12.3)	43 (14.4)	1.36 (0.87, 2.12)
50–59	4095 (15.9)	3606 (15.5)	419 (19.9)	70 (23.4)	2.49 (1.61, 3.87) ***
≥60	5680 (22.1	4477 (19.2)	1079 (51.2)	124 (41.5)	4.56 (2.95, 7.05) ***
Morbidities and comorbidities					
No reported comorbidities	17,155 (66.7)	16,059 (68.9)	956 (45.4)	140 (46.8)	1.00
Diabetes	1007 (3.9)	863 (3.7)	131 (6.2)	13 (4.3)	1.45 (1.19, 1.75) ***
Obesity	1187 (4.6)	1085 (4.7)	84 (4.0)	18 (6.0)	1.62 (1.30, 2.01) ***
Hypertension	1278 (5.0)	1088 (4.7)	169 (8.0)	21 (7.0)	1.37 (1.16, 1.63) ***
Asthma	216 (0.8)	205 (0.9)	8 (0.4)	3 (1.0)	1.06 (0.57, 1.97)
Cardiovascular disease	64 (0.2)	51 (0.2)	8 (0.4)	5 (1.7)	2.20 (1.17, 4.16) *
Renal disease	62 (0.2)	52 (0.2)	10 (0.5)	0 (0.0)	2.32 (1.14, 4.69) *
HIV	53 (0.2)	49 (0.29	2 (0.1)	2 (0.7)	1.35 (0.48, 3.84)
Immunosuppression	45 (0.2)	36 (0.2)	9 (0.4)	0 (0.0)	2.72 (1.27, 5.79) *
Other ^a^	660 (2.6)	585 (2.5)	64 (3.0)	11 (3.7)	1.43 (1.08, 1.89) *
Two comorbidities	2649 (10.3)	2167 (9.3)	422 (20.0)	60 (20.1)	1.74 (1.54, 1.97) ***
More than two comorbidities	1444 (5.6)	1162 (5.0)	254 (12.1)	28 (9.4)	1.77 (1.52, 2.06) ***
Tobacco smokers	1050 (4.1)	975 (4.2)	64 (3.0)	11 (3.7)	0.70 (0.55, 0.90) **

^a^ Tuberculosis, hemolytic anemia, neurological disease, hepatic disease, COPD, and cancer are included in this variable due to low numbers. HIV—Human Immunodeficiency Virus, COPD—Chronic Obstructive Pulmonary Disease, OR—Odds ratio, CI—Confidence interval. Data were analyzed using stepwise multivariate logistic ordinal regression. * *p* < 0.05; ** *p* < 0.01; *** *p* < 0.001.

**Table 4:** Multivariate ordinal regression analyses of factors associated with COVID-19 outcome (*n* = 25,427).

		Outcome		
	Total	Recovery	Death	OR (CI 95%)
	*n* (%)	*n* (%)	*n* (%)	
Sex				
Female	12,318 (48.4)	10,498 (20.0)	1820 (41.2)	1.00
Male	13,109 (51.6)	10,512 (50.0)	2597 (58.8)	1.54 (1.43, 1.66) ***
Age				
0–9	478 (1.9)	464 (2.2)	14 (0.3)	1.00
10–19	810 (3.2)	791 (3.8)	19 (0.4	0.80 (0.39, 1.61)
20–29	5024 (19.8)	4945 (23.5)	79 (1.8)	0.54 (0.30, 0.97) *
30–39	4892 (19.2)	4668 (22.2)	224 (5.1)	1.55 (0.89, 2.68)
40–49	4616 (18.2)	4113 (19.6)	503 (11.4)	3.56 (2.07, 6.13) ***
50–59	4045 (15.9)	3214 (15.3)	831 (18.8)	6.90 (4.02, 11.83) ***
≥60	5562 (21.9)	2815 (13.4)	2747 (62.2)	24.45 (14.30, 41.83) ***
Morbidities and Comorbidities				
No reported comorbidities	17,001 (66.9)	15,226 (72.5)	1775 (40.2)	1.00
Diabetes	989 (3.9)	694 (3.3)	295 (6.7)	1.78 (1.62, 2.09) ***
Obesity	1175 (4.6)	1008 (4.8)	167 (3.8)	2.06 (1.71, 2.48) ***
Hypertension	1258 (4.9)	867 (4.1)	391 (8.9)	1.56 (1.35, 1.80) ***
Asthma	216 (0.8)	203 (1.0)	13 (0.3)	0.84 (0.46, 1.53)
Cardiovascular disease	62 (0.2)	39 (0.2)	23 (0.5)	2.15 (1.18, 3.93) *
Renal disease	60 (0.2)	40 (0.2)	20 (0.59	4.56 (2.39, 7.71) *
HIV	51 (0.2)	42 (0.2)	9 (0.2)	2.19 (0.97, 4.96)
Immunosuppression	44 (0.2)	31 (0.1)	13 (0.3)	2.67 (1.26, 5.66) *
COPD	33 (0.1)	20 (0.1)	13 (0.1)	1.78 (0.82, 3.85)
Cancer	29 (0.0)	20 (0.1)	9 (0.2)	2.49 (1.02, 6.07) *
Neurological disease	13 (0.1)	7 (0.0)	6 (0.1)	9.51 (2.65, 34.20) **
Hepatic disease	9 (0.0)	5 (0.0)	4 (0.1)	3.63 (0.82, 16.05)
Other ^a^	464 (1.8)	382 (1.8)	82 (1.9)	1.44 (1.09, 1.89) *
Two comorbidities	2602 (10.2)	1615 (7.7)	987 (22.3)	2.18 (1.96, 2.42) ***
More than two comorbidities	1415 (5.6)	806 (3.8)	609 (13.8)	2.46 (2.16, 2.80) ***
Tobacco smokers	1034 (4.1)	884 (4.2)	150 (3.4)	0.73 (0.59, 0.89) **

^a^ Tuberculosis and hemolytic anemia are included in this variable due to low numbers. HIV—Human Immunodeficiency Virus, COPD—Chronic Obstructive Pulmonary Disease. OR—Odds ratio, CI—Confidence interval. Data were analyzed using stepwise multivariate logistic regression. * *p* < 0.05; ** *p* < 0.01; *** *p* < 0.001.

**Table 5:** Distribution of variants and their association with death as an outcome of COVID-19 (*n* = 389).

		Outcome		
Variant Tango Nomenclature	Total *n* (%)	Recovery *n* (%)	Death *n* (%)	Adjusted OR (CI 95%) ^a^
AY.20	125 (32.1)	95 (76.0)	30 (24.0)	1.00
B.1.1.519	61 (15.7)	34 (55.7)	27 (44.3)	2.23 (1.02, 4.84)*
AY.26	57 (14.7)	40 (70.2)	17 (29.8)	1.53 (0.67, 3.48)
B.1.1.7	37 (9.5)	23 (62.2)	14 (37.8)	1.72 (0.70, 4.28)
Ay.3	28 (7.2)	23 (82.1)	5 (17.9)	0.55 (0.17, 1.79)
P.1	15 (3.9)	8 (53.3)	7 (46.7)	2.30 (0.66, 7.94)
Other	66 (17.0)	48 (72.7)	18 (27.3)	1.05 (0.47, 2.33)

^a^ Adjusted with comorbidity, age, and sex. Data were analyzed using stepwise multivariate logistic regression. * *p* < 0.05.

## Data Availability

The data that support the findings of this study are available upon reasonable request from the corresponding author, but restrictions apply to the availability to these data, which were used under license for the current study, and so are not publicly available.

## Supplementary Materials

The following supporting information can be downloaded at: https://www.mdpi.com/article/10.3390/jcm12072676/s1, Table S1: Variant information (*n* = 339).
