# Supplementary material for: Sex, Age, and Comorbidities Are Associated with SARS-CoV-2 Infection, COVID-19 Severity, and Fatal Outcome in a Mexican Population: A Retrospective Multi-Hospital Study
Source: J Clin Med. 2023 Apr 3;12(7):2676. doi: 10.3390/jcm12072676 (PMC10095205; doi:10.3390/jcm12072676)
Supplement: Supplementary file 1 [file jcm-12-02676-s001.zip › jcm-2283896-Supplementary.pdf]

Journal of
Clinical Medicine

MDPI

Table S1. Variant information (n = 339).

Pango Lineage Nomenclature	n (%)
A.2.5	3 (0.76)
AY.2	1 (0.25)
AY.20	126 (31.82)
AY.25	2 (0.51)
AY.26	58 (14.65)
AY.3	29 (7.32)
AY.4	2 (0.51)
B.1	3 (0.76)
B.1.1	2 (0.51)
B.1.1.222	2 (0.51)
B.1.1.432	2 (0.51)
B.1.1.519	63 (15.91)
B.1.1.7	37 (9.34)
B.1.2	5 (1.26)
B.1.243	11 (2.78)
B.1.396	3 (0.76)
B.1.427	4 (1.01)
B.1.429	4 (1.01)
B.1.526	1 (0.25)
B.1.558	1 (0.25)
B.1.621	2 (0.51)
B.1.628	4 (1.01)
B.1.632	5 (1.26)
BA.1.1	9 (2.27)
C.37	2 (0.51)
P.1	15 (3.79)
